# Close association of kinesiophobia with physical performance in patients with systemic sclerosis

**DOI:** 10.1007/s00296-026-06072-w

**Published:** 2026-01-27

**Authors:** Atilla Uluışık, Ipek Turk, Ayşegül Yetişir, Aylin Sariyildiz, Ilke Coskun Benlidayi

**Affiliations:** 1 Department of Internal Medicine, Faculty of Medicine, Çukurova University, Adana, Turkey; 2 Division of Rheumatology, Department of Internal Medicine, Faculty of Medicine, Cukurova University, Adana, Turkey; 3 Division of Rheumatology, Department of Physical Medicine and Rehabilitation, Faculty of Medicine, Cukurova University, Adana, Turkey; 4 Department of Physical Medicine and Rehabilitation, Faculty of Medicine, Cukurova University, Adana, Turkey

**Keywords:** Scleroderma, Kinesiophobia, Mobility, Balance, Quality of life

## Abstract

The aim of this study is to identify factors associated with kinesiophobia in patients with systemic sclerosis (SSc). A total of 72 adult patients diagnosed with SSc were included in this cross-sectional study. Clinical parameters reflecting disease severity, organ involvement, and inflammatory status were recorded. Kinesiophobia level was assessed using the Tampa Scale for Kinesiophobia (TSK). The Berg Balance Scale (BBS), Y Balance Test (YBT), Timed Up and Go Test (TUG), and 10-Meter Walk Test (10MWT) were used to evaluate balance and functional capacity. Factors associated with kinesiophobia was analyzed using multiple linear regression analysis. Kinesiophobia (TSK ≥ 37) was identified in 26 patients (36.1%). Disease related parameters, including disease duration, disease activity, comorbidity burden, skin and other organ involvement (lung, gastrointestinal) were higher in patients with kinesiophobia (*p* < 0.05, for all). Patients with kinesiophobia had significantly lower BBS score, slower walking speed, longer TUG duration, and were unable to perform the YBT, compared to those without kinesiophobia (all *p* < 0.001). In the multivariable regression analysis, (β = − 1.26, CI − 1.59 to − 0.94, *p* < 0.001) and TUG (β = 1.24, CI 0.04–2.43, *p* = 0.043) showed to be associated with kinesiophobia severity independently. The multivariable model accounted for 65% of the variance in kinesiophobia (adjusted R² = 0.65). In patients with SSc, static imbalance and decreased mobilization capacity are closely associated with kinesiophobia. Monitoring these parameters should be prioritized for the management of kinesiophobia in patients with SSc.

**Supplementary Information:**

The online version contains supplementary material available at 10.1007/s00296-026-06072-w.

## Introduction

Systemic sclerosis (SSc) is a heterogeneous rheumatic disease characterized by widespread fibrosis, microvascular dysfunction, and immune activation. The disease affects many organs, primarily the skin, musculoskeletal system, lungs, cardiovascular system, and gastrointestinal system, leading to functional limitations and a significant decrease in quality of life. Complications of SSc affecting the musculoskeletal system can lead to significant limitations. Reduced joint range of motion, muscle weakness, cutaneous fibrosis, and chronic fatigue contribute to marked physical disability in affected individuals [1–3]. A recent study demonstrated that patients with SSc exhibit impaired postural balance and an increased risk of falls [4]. Such physical restrictions may foster an exaggerated perception of potential harm during movement, which over time may result in conscious avoidance of physical activity. This behavioral pattern is defined as “kinesiophobia” [5, 6].

Kinesiophobia is commonly reported in individuals with chronic conditions, such as hematological [7], cardiac [6], and and chronic pain syndromes [8]. The presence of kinesiophobia has been investigated in rheumatic diseases such as ankylosing spondylitis, rheumatoid arthritis, systemic lupus erythematosus, Sjögren’s disease, gout and sytemic sclerosis [9–13]. Patients with chronic pain deliberately avoid physical activity due to the belief that movement could cause injury or exacerbate symptoms. The clinical significance of fear of falling is not limited solely to the risk of falling; kinesiophobia is associated with muscle weakness, impaired balance function, diminished self-efficacy, and increased social isolation [14]. Fear of falling should be considered as an independent clinical problem. A recent study reported that patients with SSc exhibit higher kinesiophobia scores compared to healthy individuals [13]. However, the clinical, physical, and psychosocial factors that may associated with kinesiophobia in SSc remain unclear. Identifying these factors in this patient population could provide valuable insights for clinicians in terms of preventing or managing kinesiophobia.

The aim of this study was to compare clinical and physical factors between SSc patients with and without kinesiophobia, and to identify the parameters associated with kinesiophobia. The hypotheses to be tested in this study were as follows: (i) patients with kinesiophobia differ from those without kinesiophobia in terms of clinical characteristics and physical capacity; and (ii) clinical variables and physical capacity are related to kinesiophobia.

## Materials and methods

### Study design and patients

This research was conducted as a cross-sectional and comparative study. The study was carried out at the tertiary hospital between October 2024 and March 2025. Ethical approval was obtained from the Ethics Committee of XXX University Faculty of Medicine (Approval No: 148, Date: October 4, 2024).

The sample size was calculated a priori using the G*Power 3.1.9.2 (version 3.1.9.2; Heinrich Heine University, Düsseldorf, Germany), based on the effect size (effect size = 0.40) reported in a previous study [4]. The analysis was conducted with an effect size of 0.40, a significance level of *p* < 0.05, and a statistical power of 95% (1-β = 0.95). Accordingly, the required minimum sample size was estimated to be 70 patients.

Patients with SSc who were invited to participate in the study during routine visits and via telephone were evaluated for eligibility to be included in the study. Patients aged 18 years and older who had been diagnosed with SSc according to the 2013 ACR/EULAR classification criteria [15] and who voluntarily agreed to participate in the study were included. The exclusion criteria were as follows: (i) concomittant inflammatory rheumatologic disease, (ii) severe neurological or psychiatric disorders, (iii) any malignancy, (iv) pregnancy, and (v) history of conditions that could impair mobility, such as amputation, severe osteoarthritis, trauma, or foot ulcers. Prior to data collection, all participants received both verbal and written information regarding the study, and written informed consent was obtained from all. The study was carried out in full compliance with the principles of the Declaration of Helsinki.

### Outcome measures

#### Clinical examination

Sociodemographic information collected during the most recent clinical visit encompassed variables such as age, sex, education level, marital and employment status, parental status, smoking behavior, body mass index (BMI), disease duration, and subtype (limited or diffuse).

Gastrointestinal system involvement was determined based on the occurrence of gastroesophageal reflux, esophageal dilation, gastric antral vascular ectasia, intestinal motility disorders, or fecal incontinence [16, 17]. Interstitial lung disease was identified through typical radiologic patterns detected on high-resolution computed tomography scans [18]. Cardiac involvement was considered present when confirmed by a positive endomyocardial biopsy or, in cases clinically attributed to SSc by the attending physician, when arrhythmia, pericardial effusion, conduction disturbances, or ventricular systolic/diastolic dysfunction were demonstrated on cardiac MRI. Pulmonary arterial hypertension was diagnosed when right heart catheterization revealed a mean pulmonary arterial pressure of at least 25 mmHg together with a pulmonary arterial wedge pressure of 15 mmHg or lower [19]. Scleroderma renal crisis was diagnosed if at least two of the following criteria were met: newly developed hypertension, microangiopathic hemolytic anemia, or a progressive increase in serum creatinine concentration [20]. Cutaneous involvement was evaluated with the modified Rodnan skin score (mRSS). To minimize observer bias, a single clinician performed all assessments, classifying the skin as non-thickened, mildly, moderately, or severely thickened [21]. Disease activity was evaluated according to the updated European Scleroderma Trials and Research Group (EUSTAR) activity index (EAI), and the corresponding Valentini Disease Activity Score (VDAS) was derived from these parameters [22]. The number of digital ulcers within the past year was recorded.

Echocardiographic parameters assessed during routine follow-up, including ejection fraction and the presence of pericardial effusion, were documented. Pulmonary function test parameters, including forced vital capacity, forced expiratory volume in 1 s/forced vital capacity, and diffusing capacity of the lungs for carbon monoxide (DLCO), were also recorded. Laboratory results from the latest evaluation, including complete blood count, serum albumin, and the presence of antinuclear, anti-centromere, and anti-topoisomerase I antibodies, were documented. Details of SSc-related treatments administered within the preceding six months were documented, with specific attention to ongoing iloprost therapy. Furthermore, records included the patients’ history of falls or fractures occurring from the initial diagnosis up to the time of assessment.

#### Tampa scale for kinesiophobia (TSK)

The TSK was designed to evaluate the extent of movement-related fear that may arise from pain or previous injury. It has demonstrated good to excellent test–retest reliability, with intraclass correlation coefficients (ICC) ranging between 0.77 and 0.99, and satisfactory internal consistency, as reflected by Cronbach’s α values between 0.68 and 0.91 [23].

The instrument contains 17 statements divided into two domains: fear of movement/reinjury and avoidance beliefs. Each item is rated on a 4-point Likert scale (1 = strongly disagree, 2 = disagree, 3 = agree, 4 = strongly agree). Total scores vary from 17 to 68, where higher values indicate stronger fear-related beliefs and a greater tendency to avoid physical activity. Scores equal to or exceeding 37 are typically interpreted as the presence of kinesiophobia [6, 7]. The Turkish adaptation of the TSK was validated and shown to have strong psychometric properties by Yılmaz et al. [24].

#### Berg balance scale (BBS)

The BBS is an observational tool developed to quantitatively assess an individual’s ability to maintain postural stability during functional balance tasks. The absolute reliability of the scale was reported as 7.7 points, with an ICC value of 0.97 [25].

This assessment encompasses 14 activities, including standing unsupported, transferring between positions, standing with eyes closed, performing a tandem stance, and maintaining a single-leg stance. Each item is rated on a 5-point ordinal scale ranging from 0 (“unable to perform”) to 4 (“performs independently and safely”), resulting in a total possible score between 0 and 56. Lower scores denote greater fall risk. A total score of 0–20 reflects high risk, 21–40 indicates moderate risk, and scores of 41 or above suggest low risk of falls [26].

#### Y balance test (YBT)

The YBT is a standardized assessment used to measure dynamic balance, incorporating aspects of strength, flexibility, and proprioceptive control. Excellent test–retest reliability has been reported for this measure, with an ICC of 0.95 [27].

During testing, participants perform reach movements in three directions—anterior, posteromedial, and posterolateral—while standing on one leg at the center of a Y-shaped grid. The layout is formed by three measuring tapes arranged at 90° between the posteromedial and posterolateral axes and 135° between the anterior and posterior directions.

Participants extend the non-supporting leg along each direction, gently touching the tape with the tip of the toes without losing balance. Each direction is measured three times, and the mean reach distance (in centimeters) is recorded. These mean values are normalized to leg length and multiplied by 100 to compute the final composite score [28]. If the participant is unable to complete at least one valid trial in each of the three directions, the YBT is considered incomplete.

#### Timed up and go test (TUG)

The TUG test is a widely used, practical, and reliable tool for assessing mobility and fall risk in older adults. The TUG demonstrated good reliability, with intraclass correlation coefficients (ICC) ranging from 0.54 to 0.85 for experienced raters, and acceptable internal consistency, with a Cronbach’s α of 0.74 [29].

The test measures, in seconds, the time required for an individual to rise from a chair, walk 3 m, turn around, and return to a seated position. During the test, participants are evaluated wearing their own footwear and, if necessary, using assistive devices such as a cane or walker. A TUG time of less than 10 s is associated with normal mobility, more than 12 s is indicative of an increased risk of falling, and more than 20 s suggests severe balance impairment and a need for support [29].

#### 10-meter walk test (10MWT)

The 10MWT is used to assess walking speed as an indicator of functional mobility. It is demonstrated moderate reliability at comfortable gait speed (ICC = 0.73) and good reliability at fast gait speed (ICC = 0.82) [30].

In the 10MWT, participants were instructed to walk at their self-selected comfortable speed over a pre-measured 10-meter walkway (with assistive devices if normally used). Timing started when the participant’s foot crossed the starting line and stopped upon crossing the finish line. Two trials were performed, and the mean value was recorded in seconds [30].

#### Systemic sclerosis quality of life questionnaire (SScQoL)

The Systemic Sclerosis Quality of Life Questionnaire (SScQoL) is a disease-specific tool comprising 29 dichotomous (yes/no) items across five domains (function, mood, sleep, social life, and pain). Scores range from 0 to 29, with higher scores indicating poorer quality of life. The Turkish adaptation followed Beaton et al.’s cross-cultural guidelines [31] and was validated by Saraç et al. (2023) as a reliable and valid measure [32].

#### Scleroderma health assessment questionnaire (SHAQ)

The SHAQ expands the Health Assessment Questionnaire by adding five 10-cm visual analogue scales to assess Raynaud’s phenomenon, digital ulcers, gastrointestinal, pulmonary, and overall disease severity symptoms [33]. Scores range from 0 to 3, with higher values indicating greater disability and symptom burden. The Turkish version has been shown to be valid and reliable in patients with SSc [34].

Patient assessments (TSK, BBS, YBT, TUG, 10MWT, SScQoL, SHAQ) were performed on the same visit day, and clinical involvement and laboratory data from their last visit were recorded.

### Statistical analysis

All statistical analyses were conducted using SPSS software (version 27.0; IBM Corp., Chicago, IL, USA). Graphs were created with GraphPad Prism (version 10.4.1; GraphPad Software, LLC, San Diego, CA, USA). Data normality was evaluated through both visual inspection (histograms and Q–Q plots) and analytical testing (Kolmogorov–Smirnov test). Categorical variables are presented as counts and percentages [n (%)], whereas continuous variables are reported as mean ± standard deviation (SD) for normally distributed data or as median and interquartile range [median (IQR)] for skewed data. Between-group comparisons for categorical data were performed using Pearson’s chi-square or Fisher’s exact tests, as appropriate. Continuous data were analyzed using independent-samples t-tests for normally distributed variables or Mann–Whitney U tests when normality was not met.

Age was included as a covariate in the analysis of clinical and physical capacity variables, and pairwise comparisons were adjusted accordingly. To determine the independent associated factors of TSK scores, multiple linear regression analysis was employed. All variables showing significant differences between patients with and without kinesiophobia were initially considered as candidate associated factors for the regression analyses. Among these, conceptually overlapping variables demonstrating high intercorrelation were not entered simultaneously; instead, a single representative variable was selected (e.g., VDAS vs. VDAS category, BBS score vs. BBS category). In addition, sample size considerations were taken into account. After applying the above criteria, if more than six variables remained eligible (corresponding to an approximate ratio of 12 participants per predictor), variables with lower clinical relevance or without a plausible direct causal relationship with kinesiophobia (e.g., DLCO, medication use, or quality of life measures) were excluded. Furthermore, the YBT was not included in the regression analysis because patients with kinesiophobia were unable to perform the test, resulting in a floor effect and lack of variability. This stepwise selection strategy was used to ensure a parsimonious model and to minimize the risk of overfitting. Multicollinearity was evaluated by the variance inflation factor (VIF < 5) and tolerance (> 0.2). Regression assumptions were verified through residual analyses, with linearity and homoscedasticity visually assessed using standardized residual (ZRESID) versus predicted value (ZPRED) plots. Statistical significance was defined as *p* < 0.05 for all tests.

## Results

Systemic sclerosis patients who were invited to participate in the study during routine visits and via telephone were evaluated for eligibility to be included in the study (*n* = 94). Eight patients with overlap syndrome, three with severe psychiatric illness, two with severe neurological illness, seven with mobility-impairing illness, and two with malignancy were excluded from the study. Among these 72 patients with SSc, kinesiophobia (TSK ≥ 37) was identified in 36.1% (*n* = 26). Patients with kinesiophobia were significantly older compared to those without kinesiophobia (*p* = 0.024). Disease duration was longer and disease severity was higher in the kinesiophobia group (*p* = 0.017 and *p* = 0.002, respectively). These patients also exhibited higher comorbidity scores (*p* = 0.044), more frequent use of iloprost therapy (*p* = 0.015), and higher skin involvement scores (*p* = 0.013). Pulmonary functional capacity was more impaired in the kinesiophobia group, with significantly lower DLCO values (*p* = 0.027). Gastrointestinal involvement was more prevalent among patients with kinesiophobia (*p* = 0.036) (Table 1).

**Table 1:** Demographic and clinical characteristics stratified by TSK score in patients with systemic sclerosis

	All patients (*n* = 72)	Patients with TSK score ≥ 37 (*n* = 26)	Patients with TSK score < 37 (*n* = 46)	*p* value
Age (years)*	55 ± 11.6	59.1 ± 11.1	52.7 ± 11.4	0.024
Sex (female)**	66 (91.7)	25 (96.1)	41 (89.1)	0.300
Employment status**				
Employed	4 (5.6)	0 (0)	4 (8.7)	0.122
Unemployed	68 (94.4)	26 (100)	42 (91.3)	
Smoking**				
Never	52 (72.2)	18 (69.2)	34 (73.9)	
Current smoker	7 (9.7)	3 (11.5)	4 (8.7)	0.896
Former smoker	13 (18.1)	5 (19.2)	8 (17.4)	
Smoking (pack/years)	0 (4.5)	0 (6.3)	0 (3)	0.772
BMI (kg/m²)*	26.83 ± 5.12	27.97 ± 5.86	26.19 ± 4.58	0.157
History of falls**	13 (18.1)	5 (19.2)	8 (17.4)	0.845
History of fractures**	5 (6.9)	2 (7.7)	3 (6.5)	0.851
ANA**	69 (95.8)	25 (95.2)	44 (95.7)	0.919
SSA**	14 (19.4)	6 (23.1)	8 (17.4)	0.558
SSB**	2 (2.8)	1 (3.8)	1 (2.2)	0.678
ACA positivity**	20 (27.8)	6 (23.1)	14 (30.4)	0.503
Anti-Scl70 positivity**	44 (61.1)	17 (65.4)	27 (68.7)	0.576
CRP (mg/L)	4 (6)	5.5 (9.3)	3.7 (4.4)	0.103
Disease duration, (years)	10 (9)	12.5 (8.3)	8 (10)	0.017
Type**				
Diffuse	27 (37.5)	11 (42.3)	16 (34.8)	
Limited	45 (62.5)	15 (57.7)	30 (65.2)	0.526
VDAS	2.5 (4.5)	4.3 (4.5)	1.5 (3.6)	0.002
VDAS-Category**				
≤3 puan	42 (58.3)	10 (38.5)	32 (69.6)	0.010
> 3 puan	30 (41.7)	16 (61.5)	14 (30.4)	
CCI score	3 (2)	4 (3)	3 (2)	0.044
Medications**				
None	15 (20.8)	5 (19.2)	10 (21.7)	
Azathioprine	14 (19.4)	6 (23.1)	8 (17.4)	
MMF	22 (30.6)	9 (34.6)	13 (28.3)	
DMARD	7 (9.7)	2 (7.7)	5 (10.9)	
MMF + DMARD	12 (16.7)	3 (11.5)	9 (19.6)	
Azathioprine + DMARD	2 (2.8)	1 (3.9)	1 (2.2)	0.910
Iloprost**	39 (54.2)	19 (73.1)	20 (43.5)	0.015
mRSS*	15.1 ± 7.8	18.1 ± 6.3	13.4 ± 8.1	0.013
Active DU**	28 (38.9)	12 (46.2)	16 (34.8)	0.342
Number of DU in the last year	2 (3)	2 (3)	1 (2)	0.072
ILD**	49 (68.1)	20 (76.9)	29 (63)	0.225
PAH**	10 (13.9)	6 (23.1)	4 (8.7)	0.090
PAP, (> 40 mmHg)**	9 (12.5)	5 (19.2)	4 (8.7)	0.194
FVC (%)*	89.6 ± 26.8	85.7 ± 29.2	91.8 ± 25.5	0.055
FEV1/FVC	79 (7)	79 (7)	79.5 (7)	0.661
DLCO, (ml/min/mmHg)*	59.7 ± 21	52.3 ± 22	63.9 ± 19.5	0.027
Cardiac involvement**	10 (13.9)	5 (19.2)	5 (10.9)	0.324
EF (%)	65 (5)	65 (4.5)	65 (5)	0.744
Pericardial effusion**	4 (5.6)	2 (7.7)	2 (4.3)	0.552
SSc renal crisis**	1 (1.4)	0 (0)	1 (2.2)	0.449
GERD**	50 (69.4)	22 (84.6)	28 (60.9)	0.036
SScQoL	14.5 (13.8)	23.5 (9)	10.5 (12)	< 0.001
SHAQ	0.92 (1.22)	1.92 (0.68)	0.69 (0.54)	< 0.001

Values are presented in: *: mean ± standart deviation, **: n (%), and others: median (IQR)

SD: Standard deviation; IQR: Interquartile range; ANA: Antinuclear antibody; SSA: Anti-Sjögren antibody A; SSB: Anti-Sjögren antibody B; ACA: Anti-centromere antibody; CRP: C-reactive protein; CCI: Charlson comorbidity index; VDAS: Valentini Disease Activity Score; MMF: Mycophenolate mofetil; DMARD: Disease-modifying antirheumatic drug; SSc: Systemic sclerosis; mRSS: Modified Rodnan Skin Score; DLCO: Diffusing capacity of the lung for carbon monoxide; FVC: Forced vital capacity; FEV1: Forced expiratory volume in 1 s; DU: Digital ulcer; ILD: Interstitial lung disease; PAH: Pulmonary arterial hypertension; PAP: Pulmonary artery pressure; EF: Ejection fraction; GERD: Gastroesophageal reflux disease; SScQoL: Systemic Sclerosis Quality of Life Questionnaire; SHAQ: Scleroderma Health Assessment Questionnaire

Table 2 indicates that patients with kinesiophobia had significantly worse physical capacity than those without. Their BBS scores were markedly lower (*p* < 0.001), walking speed in the 10-MWT was slower (*p* < 0.001), and TUG times were longer (*p* < 0.001). Moreover, none of the patients with kinesiophobia could perform the YBT, while the majority without kinesiophobia succeeded (*p* < 0.001).

**Table 2:** Comparison of balance and gait-related physical capacities in patients stratified by kinesiophobia scores

	All patients (*n* = 72)	Patients with TSK score ≥ 37 (*n* = 26)	Patients with TSK score < 37 (*n* = 46)	*p* value
BBS scores*	53 (18.3)	32.5 (16.3)	55 (1.3)	< 0.001
BBS category				
Low	46 (63.9)	1 (3.8)	45 (97.8)	
Moderate	20 (27.8)	19 (73.1)	1 (2.2)	< 0.001
High	6 (8.3)	6 (23.1)	0 (0)	
10-MWT, (m/s)*	1 (0.4)	0.7 (0.3)	1.1 (0.2)	< 0.001
TUG (s)*	10 (4.6)	14 (4.3)	9 (2)	< 0.001
Y-Balance test performers	38 (52.8)	0 (0)	38 (82.6)	< 0.001
Y-Balance test (Left leg)*				
Anterior	49 (60.3)	0 (0)	58.8 (12.5)	
Posteromedial	52 (69.4)	0 (0)	64.7 (15.1)	
Posterolateral	0 (50)	0 (0)	6.2 (54.5)	
Total	37.5 (56.4)	0 (0)	55.2 (20.5)	< 0.001
Y-Balance test (Right leg)*				
Anterior	47 (59.5)	0 (0)	57 (13)	
Posteromedial	49.8 (63)	0 (0)	59.2 (17.6)	
Posterolateral	0 (49.8)	0 (0)	46.7 (54.9)	< 0.001
Total	37.1 (56.6)	0 (0)	53.9 (21.5)	

Values are presented in: *: median (IQR) and others: n (%)

SSc: Systemic sclerosis; TSK: Tampa Scale for Kinesiophobia; BBS: Berg Balance Scale; 10-MWT: 10-meter walk test; TUG: Timed Up and Go test; IQR: Interquartile range

**Fig. 1:**
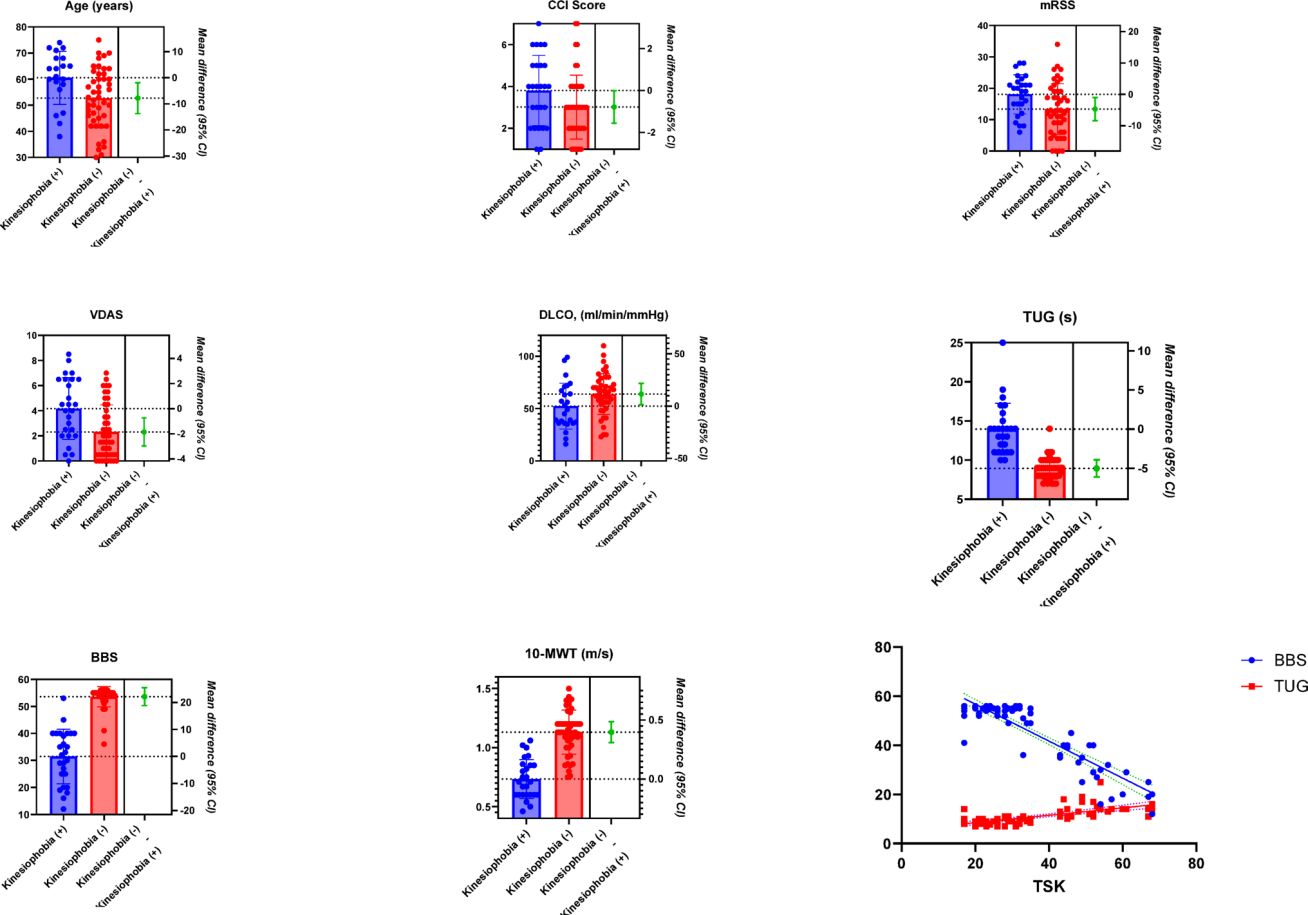
The continous data that showed significant differences in pairwise comparisons and significant effects in multivariable regression analysis. CCI: Charlson comorbidity index; mRSS: Modified Rodnan Skin Score; VDAS: Valentini Disease Activity Score; DLCO: Diffusing capacity of the lung for carbon monoxide; TSK: Tampa Scale for Kinesiophobia; BBS: Berg Balance Scale; 10-MWT: 10-meter walk test; TUG: Timed Up and Go test

The univariable linear regression adjusted for age and sex, identified several significant associated factors of kinesiophobia, including disease duration (B = 0.53, *p* = 0.021), VDAS (B = 8.20, *p* = 0.015), mRSS (B = 0.43, *p* = 0.048), BBS score (B = − 0.91, *p* < 0.001), 10-MWT (B = − 32.41, *p* < 0.001), and TUG (B = 2.44, *p* < 0.001). However, in the multivariable linear regression adjusted for age and sex, only BBS score (B = − 1.26, *p* < 0.001) and TUG time (B = 1.24, *p* = 0.043) remained independent associated factors of kinesiophobia. The final multivariable model accounted for 65% of the variance in kinesiophobia (adjusted R² = 0.65), suggesting a strong explanatory power (Table 3). Diagnostic analyses indicated that the assumptions of linear regression were met, with no multicollinearity (variance inflation factor < 5 and tolerance > 0.20), homoscedastic residuals, and an approximately normal distribution of the age- and sex-adjusted unstandardized residuals (Supplementary Appendix). The continous data that showed significant differences in pairwise comparisons and significant effects in multivariable regression analysis are presented in Fig. 1.

**Table 3:** Univariable and multivariable regression analyses of clinical and physical capacity parameters associated with TSK scores in patients with systemic sclerosis, adjusted for age and sex

	Univariable regression				Multivariable regression		
	B (SE)	95% CI for B	*p* value	Adjusted *R*^2^	B (SE)	95% CI for B	*p* value
Disease duration	0.53 (0.23)	0.08 to 0.99	0.021	0.06	− 0.19 (0.16)	− 0.50 to 0.13	0.240
VDAS	8.20 (3.20)	1.65 to 14.75	0.015	0.07	− 0.17 (2.47)	− 5.11 to 4.77	0.944
mRSS	0.43 (0.21)	0.01 to 0.85	0.048	0.04	− 0.02 (0.15)	− 0.31 to 0.28	0.902
BBS	− 0.91 (0.08)	− 1.07 to − 0.75	< 0.001	0.64	− 1.26 (0.16)	− 1.59 to − 0.94	< 0.001
10-MWT	− 32.41 (5.22)	− 42.83 to − 21.99	< 0.001	0.35	4.50 (7.84)	− 11.16 to 20.17	0.568
TUG	2.44 (0.42)	1.60 to 3.29	< 0.001	0.31	1.24 (0.60)	0.04 to 2.43	0.043
					Adjusted R^2^ = 0.65		

B: Unstandardized coefficients, SE: Standard error, Beta: Standardized coefficients; mRSS: Modified Rodnan Skin Score; ESR: Erythrocyte sedimentation rate; VDAS: Valentini Disease Activity Score; BBS: Berg Balance Scale; 10-MWT: 10-meter walk test; TUG: Timed Up and Go test

## Discussion

This study aimed to elucidate the clinical and physical factors associated with kinesiophobia in patients with SSc, demonstrated that those with kinesiophobia had longer disease duration, higher disease activity, more pronounced skin involvement, and gastrointestinal manifestations. In addition, static and dynamic balance performance and functional mobility were reduced in patients with kinesiophobia compared to those without. Notably, a close relationship has been found between impaired static balance and mobility and kinesiophobia. By identifying the clinical and physical factors associated with kinesiophobia in SSc, this study contributes valuable evidence to the current literature.

Kinesiophobia has been increasingly investigated across various patient populations and disease contexts [5–7]. Previous studies have shown that kinesiophobia is frequently observed in patients with rheumatic diseases including SSc [8–13]. The present study identified that more than 1/3 of SSc population (36.1%) had kinesiophobia. Compared to the study by Pehlivan et al., our study included a larger number of patients with SSc and utilized objective tests for physical capacity assessment: BBS, YBT, TUG, and 10MWT. Additionally, quality of life was assessed using the SScQoL and clinical manifestations have been examined in more detail [13].

Kinesiophobia is known to reduce physical activity levels and adversely affect quality of life [5, 14]. In the present study, consistent with previous findings, patients with SSc who exhibited higher levels of kinesiophobia demonstrated lower quality of life and poorer overall health status compared to those without elevated kinesiophobia. In several chronic diseases, the severity of clinical symptoms can influence their interest in or willingness to engage in physical activity [35, 36]. Previous studies have shown that, among the clinical parameters influencing kinesiophobia scores, disease severity appears to be more prominent than other inflammatory or disease-specific factors [37]. Comorbidities and biochemical parameters have also been shown to be associated with kinesiophobia [36]. In the current study, skin and organ involvement as well as the VDAS category (≥ 3) was more frequently observed in SSc patients who experience kinesiophobia. However, the regression analysis did not provide direct evidence that these parameters influence kinesiophobia. Instead, certain physical parameters—which are usually overlooked in daily clinical practice—appeared to affect kinesiophobia level independently. This study revealed that dynamic and static balance, functional mobility, and gait duration were worse in SSc patients with kinesiophobia compared to those without. In our study, it was noteworthy that none of the patients in the kinesiophobia group could complete the Y Balance Test. SSc patients have been shown to have worse functional balance, dynamic balance, static balance, and proprioception compared to a healthy control group [38]. Many factors, such as physical limitations, joint problems, muscle weakness, impaired proprioception, balance disorders, and fear of movement, may have contributed to this situation. Impaired dynamic balance may have led to kinesiophobia, or the presence of kinesiophobia may have contributed to worse dynamic balance. There appears to be a bidirectional relationship between kinesiophobia and dynamic balance. Considering the existing literature, this finding is not surprising for researchers. In several rheumatic conditions such as knee osteoarthritis [28], rheumatoid arthritis [39], and fibromyalgia [40], significant associations between physical fitness parameters and kinesiophobia have been demonstrated. Musculoskeletal involvement is more frequent than expected in patients with SSc and, although the prognosis of the disease largely depends on visceral involvement, it remains one of the major causes of disability. Among the clinical manifestations of musculoskeletal involvement, arthralgia and muscle weakness are considered the main contributors to balance impairment and gait dysfunction [41] As the most important finding of the study, these indicators of physical function were found to be more strongly associated with kinesiophobia than clinical and laboratory parameters related to the disease. We recommend that clinicians, who typically focus on monitoring clinical parameters, should also assess factors reflecting physical status (i.e., static balance and gait speed) for the management of kinesiophobia.

This study has certain limitations. First, although several physical and clinical parameters were evaluated, variables reflecting the social and psychological status of patients and their potential impact on kinesiophobia were not investigated. Our study focused on the ‘physical’ aspect of the biopsychosocial framework, and the kinesiophobia-related factors in the regression analysis may change when psychosocial factors are included. Second, the sample size did not allow the inclusion of all variables (those found significant in pairwise comparisons) in the regression analysis. On the other hand, in our study, functional mobility and dynamic balance were evaluated using objective tools, and their relationship with kinesiophobia was investigated. Considering these limitations, the findings of this study may be tested in future research designed within a biopsychosocial framework and conducted with a larger sample size that enables advanced or subgroup analyses.

In conclusion, this study highlights that although there are differences in various clinical parameters between SSc patients with and without kinesiophobia, physical status indicators, particularly static balance and gait speed, are more closely associated with kinesiophobia. These findings emphasize the importance of incorporating physical capacity assessments into daily clinical practice and suggest that multidisciplinary approaches targeting musculoskeletal function may be essential for the effective management of kinesiophobia in SSc.

## Supplementary Information

Below is the link to the electronic supplementary material.

Supplementary Material 1

## Data Availability

The datasets gathered during the preparation of this manuscript are available from the corresponding author upon reasonable request.
