# Supplementary material for: Close association of kinesiophobia with physical performance in patients with systemic sclerosis
Source: Rheumatol Int. 2026 Jan 27;46(2):36. doi: 10.1007/s00296-026-06072-w (PMC12847095; doi:10.1007/s00296-026-06072-w)

**Supplementary Appendix:** Diagnostic evaluation of linear regression assumptions

**Table S1** Collinearity statistics

| Model | | Collinearity Statistics | |
| --- | --- | --- | --- |
|  |  | Tolerance | VIF |
| 1 | (Constant) |  |  |
|  | Disease_duration | ,799 | 1,251 |
|  | VDAS | ,661 | 1,513 |
|  | mRSS | ,752 | 1,330 |
|  | BBS | ,236 | 4,246 |
|  | Ten_MWT | ,236 | 4,242 |
|  | TUG | ,256 | 3,901 |

**Figure S1** Diagnostic plots for linear regression assumptions


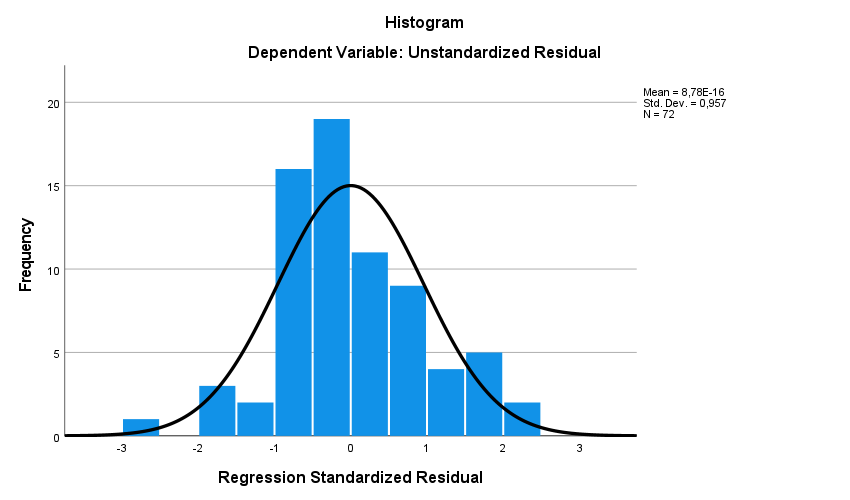


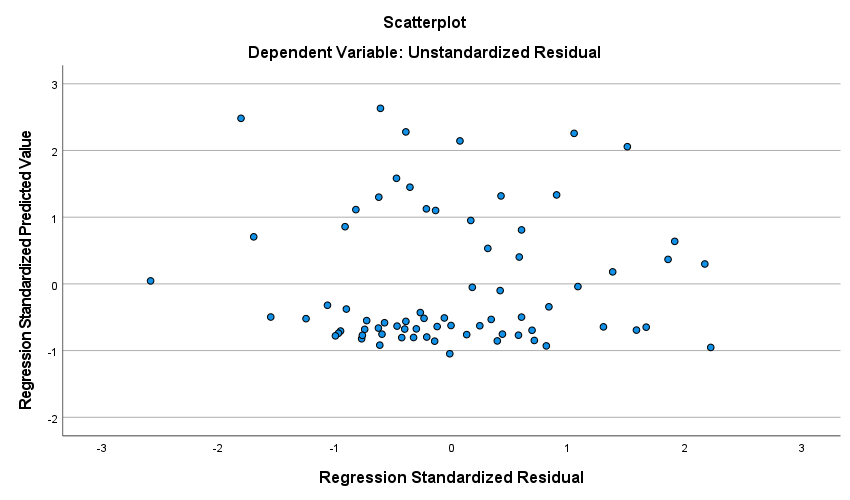


**Figure S2** Normal Q–Q plot of age- and sex-adjusted unstandardized residuals


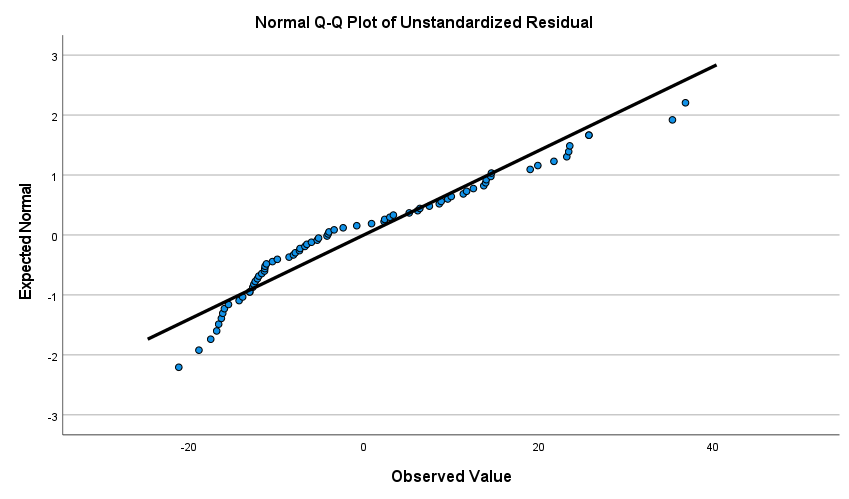

Supplement: Supplementary file 1 — Supplementary Material 1 [file 296_2026_6072_MOESM1_ESM.docx]
